# Analysis of severe fever with thrombocytopenia syndrome cluster in east China

**DOI:** 10.1186/s12985-023-02155-3

**Published:** 2023-09-01

**Authors:** Tao Liu, Nannan Zhang, Haiwen Li, Shuting Hou, Xiuwei Liu

**Affiliations:** 1 Department of Infectious Disease Control, Yantai Center for Disease Control and Prevention, 17 Fuhou Rd, Laishan District, Yantai, Shandong Province P. R. China; 2 Department of Infectious Disease Control, Zhaoyuan Center for Disease Control and Prevention, Yantai, Shandong Province P. R. China

**Keywords:** Tick-borne transmission, SFTS, China, bunyavirus

## Abstract

**Background:**

Severe fever with thrombocytopenia syndrome (SFTS) is a common tick-borne, natural focal disease. SFTS virus (SFTSV) transmission can occur between family members through close contact with an infected patient. In this study, we explored the possible transmission route of an outbreak cluster in east China.

**Method:**

A case-control study was carried out to analyze the potential risk factors for person-to-person transmission. Bunia virus was detected by IgM antibody, enzyme-linked immunosorbent assay, and reverse transcription polymerase chain reaction. Chi-square, univariate, and multivariate analyses were performed to calculate the association of possible risk factors for SFTSV transmission.

**Results:**

Two patients had a clear history of blood and aerosols contact, and one may be exposed to aerosols in a closed environment. Five close contacts of the Index patient were IgM-positive and three were IgM and SFTSV RNA positive. Exposure to a poorly ventilated space where the corpse was stored (χ^2^ = 5.49, *P* = 0.019) and contact with the Index patient’s contaminated items (χ^2^ = 15.77, *P* < 0.001) significantly associated with SFTSV infection.

**Conclusion:**

We suspect that the cluster outbreak was possibly a person-to-person transmission of SFTSV, which may have been transmitted by directly contacting with blood of SFTS patient. The propagation of aerosols in closed environments is also an undeniable transmission.

## Introduction

Severe fever with thrombocytopenia syndrome (SFTS) is an emerging infectious disease that was first reported in 2009[1]. This disease was later reported in more than 17 Chinese provinces [2]. SFTS patients also be reported in South Korea, Japan and United Arab Emirates [3–5]. The United States reported two patients and isolated a virus named Heartland Virus [6]. Liu et al. reported that the case-fatality rates of SFTS range from 2.5 to 30%, which would pose threat to public health if it ever becomes widespread [7].

The disease etiological agent named SFTS virus (SFTSV) which was firstly isolated from patients with fever, thrombocytopenia, leukocytopenia, and multiorgan dysfunction [1]. Currently, SFTSV has been assigned to genus Bandavirus in family Phenuiviridae of Bunyavirales by International Committee on Taxonomy of Viruses (ICTV)[8]. SFTSV belonged to segmented, negative-strand RNA that contains large (L), medium (M), and small (S) segments [1]. The L segment is encoded by RNA-dependent RNA polymerase (RdRp), which can act as the viral transcriptase/replicase. The M segment encoded by 1073 amino acids glycoprotein (Gn and Gc), and the S segment is an ambisense RNA that encodes the nucleocapsid protein (NP) and nonstructural protein (NSs) [9]. The pathogenic mechanism of SFTSV has not been fully defined, current studies suggest that SFTSV can prevent the immune response of the host. Benjamin et al.[10] found that NSs can inhibit the interferon (IFN) responses and suppress viral replication. Cytokine storm has also been noted to be related to the severity of SFTS [11]. In addition, SFTSV has shown to bind to platelets, which could lead to the decrease in platelet count due to the recognition of macrophages and phagocytosed for SFTSV [12].

The symptoms of SFTS include high fever, thrombocytopenia, leucopenia, gastrointestinal and central nervous system symptoms. Severe cases may lead to multiple organ failure or death [13, 14]. Currently, no vaccine or treatment is available to treat SFTS. Ribavirin and supportive treatment, including treatment with plasma exchange, intravenous immunoglobulin, and corticosteroid had been reported to be benefit for SFTS [15]. However, it is difficult to determine which treatment strategy is effective against SFTS due to insufficient data.

Tick bites are the main transmission route for SFTSV, followed by contact with confirmed patients’ infected blood or bodily fluid [16, 17]. The incubation period of SFTS through human-to-human transmission is 3–15 days, with a median of 10 days [18]. In addition, Moon et al. found that SFTSV was transmitted from person to person through aerosols during aerosol-generating procedures [19]. The presence of SFTSV RNA has been reported in semen, suggesting that SFTSV is capable of sexual transmission [20]. SFTS has been reported to have spread among family members [21]. Huang et al.[22] reported a cluster outbreak in six suspected SFTS patients, and the authors concluded that exposure to blood was the primary risk factor. We report a family cluster analysis of SFTS in Zhaoyuan, Shandong province in China. The cause, transmission mode, and risk factors for the outbreak were analyzed.

## Methods

### Subjects

The Index patient developed a fever on June 12, died on June 21, and was cremated on June 22. Anyone who lived, worked, talked, or physically contacted with the Index patient from June 12 to June 22 were defined as close contact. Investigators examined 49 close contacts and 36 others from the same village who did not meet the definition of a close contact in the period from diagnosis to cremation were firstly included in epidemical investigation for case research when the Index patient was diagnosed. Any person with a positive test for SFTSV nucleic acid was defined as an active SFTS patient. Those with positive SFTSV IgM antibodies were defined as SFTS-infected individuals.

### Laboratory analysis

Venous blood (5 mL) was collected from close contacts and other villagers. SFTSV RNA was tested by reverse transcription polymerase chain reaction (RT-PCR), and specific IgM antibodies were detected by a commercial enzyme-linked immunosorbent assay kit according to the manufacturer’s instructions. Presence of the virus was defined by a cycle threshold value < 35 for RT-PCR and optical density above the cut-off value.

Between June 12 and June 22, At the same time, on the voluntary and informed basis, we collected 5 ml of venous blood from 36 villagers in the same village location as of the Index case consented to blood sampling as unexposed healthy controls. None of these volunteers had any contact with the Index patient.

### Epidemiological investigation

Two case-control studies were designed, we first divided the 49 close contacts of the Index patient into cases group (3 SFTSV nucleic acid positive persons) and controls group (46 SFTSV nucleic acid negative persons) based on the SFTSV test. Then we divided the 49 close contacts of the Index patient into cases group (8 IgM positive persons) and controls group (41 IgM negative persons) based the IgM examination. SFTSV-infected persons were close contacts of the Index patient. Controls were close contacts who were not infected. Close contacts of the Index patient were evaluated for the potential risk factors for person-to-person transmission of the cluster of SFTS.

Data were collected in-person by well-trained interviewers. A structured questionnaire included questions on demographics (age, gender, ethnic group, home address, and occupation); living environment (landform, poultry, ventilation, animals and house rats); exposure history (travel history, tick bites, contact with Index patient’s contaminated items, and contact with SFTS patients); contacts with animals (animal species and types of vectors); use of personal protection. Exposure history in particular was obtained by subject recall for the month preceding the data collection.

### Field collection of ticks

Ticks were collected from nearby fields and hills from the location of the Index patient based on the epidemiological survey conducted from July 13 to July 15. A white flannel cloth drape (1 m^2^) was dragged over the plant-life to capture feeding ticks. Ticks were then sorted according to species.

### Statistical analysis

Chi-square tests, univariate, and multivariate logistic regression were conducted to calculate the odds ratios (OR) and 95% confidence intervals (95% CI). All statistical calculations were performed using SPSS Statistics for Windows, version 11.0 (SPSS Inc., Chicago, Ill., USA) and *P* < 0.05 was considered significant.

## Results

### Index patient

The Index patient was a 57-year-old woman, who lived with her husband in the center of a township in Zhaoyuan County. Their yard lacked shrubbery or domestic animals. She had a history of exposure to ticks during farm work. On June 12, 2020, the patient reported fever, chills, nausea, and vomiting. She self-medicated some ibuprofen granules and cephalothin at home. Her symptoms worsened and she was admitted in Hospital A on June 17. Laboratory tests revealed a white blood cell (WBC) count of 1.5 × 10^9^/L (lower boundary of normal 4.0 × 10^9^/L), neutrophils 46.6% of total WBC, and a platelet counts of 65 × 10^9^/L (lower boundary of normal 100 × 10^9^/L). On the same day, she was transferred to Hospital B, an infectious disease center, where she was diagnosed with SFTS. On June 19, a positive RT-PCR result for SFTSV was reported by the Yantai Center for Disease Control and Prevention. Subsequently, her WBC and platelet counts declined below admission levels (1.17 × 10^9^/L and 55 × 10^9^/L, respectively). Her creatine kinase (CK) level was > 2000 U/L, lactase dehydrogenase (LDH) level was > 900 U/L, and urea level was 9.07 mmol/L. She lost consciousness on June 19, and her WBC and platelets counts further decreased to 1.48 × 10^9^/L and 58 × 10^9^/L, respectively. The CK value, acetic acid dehydrogenase value, AST, ALT, PTT, creatinine, and urine protein were 9670 U/L, 1719 U/L, 193.9 U/L, 193.9 U/L, 53.6 s, 1719 U/L and 3+, respectively. The clinician’s diagnosis showed that organ impairment in the heart, liver and kidneys. The patient experienced bleeding in multiple parts of the body, resulting in blood contamination of clothing and utensils. Considering her clinical status, her relatives elected to stop treatment and brought her home on June 21. The patient died at the same day. Her body was cremated on June 22. Until the end of the funeral, all items she used from illness to death were discarded. And her home was disinfected.

### Epidemiological investigation for cases in the cluster

**Fig. 1:**
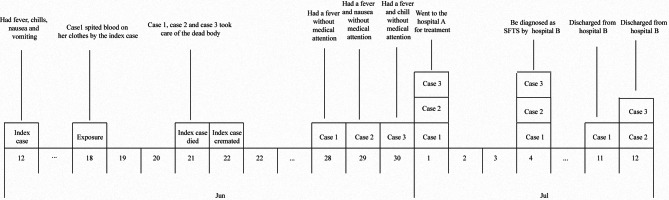
Timeline of disease onset dates for a cluster of four SFTS patients. Epidemic curve shows progression of critical symptoms during the Index patient’s illness, and the onset of SFTS in the three clustered patients, of which the onset date ranged from 7 to 13 days after exposure to the dead body of the Index patient

Of those who were in close contact with the Index patient, we found 3 SFTS cases with RT-PCR positive results and typical symptoms of SFTS. These three cases were diagnosed as secondary patients from 7 to 13 days after the Index patient died (Fig. 1). The demographic characteristics of the four patients are displayed in Table 1. Another five cases were identified with asymptomatic SFTS infection that were only IgM-positive.

**Table 1:** Epidemiologic features of a cluster of 4 patients with SFTS

Features	Index patient	Case 1	Case 2	Case 3
Sex	Female	Female	Male	Male
Age (years)	57	32	30	62
Occupation	Farmer	full-time housewife	office worker	Farmer
Tick-bite history	Yes	No	No	No
Underlying disease	No	No	No	No
Date of onset	Jun12, 2020	Jun 28, 2020	Jun 29, 2020	Jun 30, 2020
Incubation period (days)	NA	NA	8	9
Course of disease (days)	10	14	14	13
Outcome	Death	Discharge	Discharge	Discharge

#### Case 1

was the Index patient’s daughter who was a full-time housewife. She does not live in the same village as the Index patient. Case 1 was accompanied with the Index patient in the hospital from June 12 to June 21. She wore personal protection most of time, such as wearing masks and gloves during the processes and reported no contact with the blood, bodily fluids or other secretions from the Index patient. Unfortunately, she was exposed to vomiting blood from the Index case on June 18. She reported participation in funeral affairs, cleaning and dressing the patient before cremation while wearing personal protective gears. She had fever (39.5 ºC), chills, joint and muscle aches on June 28 and was treated at home with some ibuprofen granules and cephalothin. She was diagnosed with suspected SFTS and was admitted to Hospital A on July 1. The laboratory tests revealed thrombocytopenia and leukopenia. Her diagnosis was updated to SFTS and she was transferred to Hospital B on July 4. She was discharged on July 11 after treatment at Hospital B.

#### Case 2

was the Index patient’s younger son who was an office worker in Beijing until June 21. He reported participation in funeral affairs, cleaning and dressing the patient before cremation while wearing personal protective gears. However, he was in direct contact with the blood on the deceased patient on June 21. He stayed at home of the Index patient and participated in all activities from June 22 to June 28. On June 29, he developed a fever (38 ºC) with general fatigue and went to the clinic at Hospital A for medical care. The laboratory results showed that he had thrombocytopenia and leukopenia. He was then transferred to Hospital B on July 4 where he was diagnosed with SFTS. He remained there until his discharge on July 12.

#### Case 3

was the Index patient’s husband. He reported not directly exposed to the blood of the Index patient. However, he participated in funeral affairs and cleaned the Index patient’s remains while wearing personal protection. He reported dizziness and severe nausea, and was admitted to Hospital A for medical care on June 30. Laboratory tests revealed leukopenia. Craniocerebral computed tomography (CT) showed multiple sites of cerebral ischemia, infarction, and soft lesions. He quickly developed a high fever of 39 ºC. His WBC count further decreased on July 2. He was transferred to Hospital B where he was diagnosed with SFTS on July 4. He was discharged on July 12.

None of them reported a history of tick bite or animal contact within two weeks of the onset of SFTS. Case 1 and Case 2 came into contact with the blood of the Index patient with personal protection. Case 3 had not direct history of blood exposure. All three patients had been exposed for a long time to a closed environment where the patient’s corpse and her blood contaminated clothing is stored. The family in this case sealed the house from the yard outside using glass and other materials to maintain warmth and cleanliness. Moreover, the windows of the house could not be opened, resulting in a poorly ventilated environment.

### Clinical characteristics

As shown in Table 2, the body temperature of the four patients with SFTS ranged from 38 ºC to 39 ºC, and they had typical SFTS clinical features, such as gastrointestinal symptoms, thrombocytopenia, and leukocytopenia. The Index patient had skin silt ecchymosis and respiratory failure, and died of disseminated intravascular coagulation. The three secondary cases all recovered and appeared to have no long-term injury on follow-up.

**Table 2:** Clinical and laboratory information of the 4 patients with SFTS in the cluster

Features	Index patient	Case1	Case2	Case3
Clinical symptoms				
Fever	Yes	Yes	Yes	Yes
shiver	Yes	No	No	Yes
Nausea	Yes	No	Yes	No
Vomiting	Yes	No	Yes	No
Myalgia	No	No	No	Yes
Weak	Yes	No	No	No
Skin silt ecchymosis	Yes	No	No	No
WBC^1^ (×10^9^)	1.17	2.6	2.5	2.9
PLT^2^ (×10^9^)	55	61	85	98
ALT^3^(U/L)	193.9	NA	NA	normal value
AST^3^(U/L)	193.9	NA	NA	normal value
LDH^3^(U/L)	＞900U/L	NA	766	normal value
CK^3^(U/L)	> 2000U/L	NA	162	normal value

WBC, with blood cell;PLT, platelet;ALT,alanine aminotransferase;AST,aspartate aminotransferase;LDH,lactate dehydrogenase;CK,creatine kinase

### Virus detection in close contacts and unexposed villagers

We examined 49 close contacts of the Index patient on July 4, 2020. Five subjects tested positive for SFTSV-specific IgM. Of these, three persons tested positive for both IgM and viral RNA. The SFTSV-specific IgM positivity rate was accounted for 16.3% (8/49).

Thirty-six unexposed residents from the same village as the Index case consented to blood testing between July 13 to 15. Nucleic acid and antibody test results were negative for all the volunteers.

### Investigation of the natural environment and biological vectors

Tick monitoring showed that *Haemaphysalis longicornis*, *Rhipicephalus microplus*, *Boophilus* miniature ticks, and other ticks are distributed in Zhaoyuan City. *Haemaphysalis longicornis*, the major vector, was the most abundant human-biting tick species. We captured 45 *Haemaphysalis* long horned ticks near the Index patient’s workplace from July 13 to 15, and none of these tested positive for SFTSV RNA.

### Risk factors of person-to-person transmission of SFTSV

A case-control study of the 49 close contacts of the Index patient was conducted to explore the factors contributing to person-to-person transmission of SFTSV. Three STFS cases were compared to 46 uninfected controls (Table 3). No risk factors were found to be associated with SFTS.

**Table 3:** Univariate logistic regression analyses of potential risk factors among person-to-person transmission of SFTSV

Exposure factors	Case n = 3(%)	Control n = 46(%)	OR	*95%CI*	*P*
Attending to the index patient’s funeral	3 (100.00)	28 (60.86)	0.00	-^1^	1.00
The poor ventilationt where the corpse was stored	3 (100.00)	24 (52.17)	0.00	-^1^	1.00
Talked face to face	2 (66.67)	20 (46.47)	0.39	0.033–4.55	0.45
The index patient’s contaminated items	3 (100.00)	13(28.26)	0.00	-^1^	1.00
The index patient’s vomitus	1 (33.33)	0 (0.00)	0.00	-^1^	1.00
The index patient’s excreta	1 (33.33)	1 (2.17)	0.044	0.002-1.00	0.50
The index patient’s blood or body fluids	2 (66.67)	7 (15.22)	0.090	0.007–1.13	0.062
Rats in the households	0 (0.00)	4 (8.70)	0.00	-^1^	1.00
Domestic animals	0 (0.00)	7 (15.22)	0.00	-^1^	1.00
Weeds and shrubs in household	0 (0.00)	4 (8.70)	0.00	-^1^	1.00
Wild animals in household or working place	0 (0.00)	6 (13.04)	0.00	-^1^	1.00
Worked in the field	1 (33.33)	28 (60.87)	0.37	0.027–3.81	0.32
Weeds and shrubs in workplace	1 (33.33)	23 (50.00)	0.50	0.042–5.91	0.58

^1^Infifinite or not able to be calculated

A second case-control analysis compared 8 IgM-positive cases and 41 IgM-negative controls (Table 4). No risk factors were found to be related to SFTS upon univariate analysis (Table 4), but analysis by χ^2^ test showed that cases were more likely to be exposed to poor ventilation where the corpse was stored (χ^2^ = 5.49, *P* = 0.019) and to be in contact with the Index patient’s contaminated items (χ^2^ = 15.77, *P* < 0.001) (Table 4).

**Table 4:** Univariate analyses and χ^2^ test of potential risk factors of person-to-person transmission of serum samples IgM antibodies positive SFTSV

Exposure factors	Cases n = 8 (%)	Controls n = 41 (%)	Univariate analyses			χ^2^ test	
			OR (95%CI)	*P*		χ^2^	*P*
Attending to the index patient’s funeral	8 (100.00)	23 (56.10)	0.00(-)^3^	1.00		3.57	0.059^1^
The poor ventilation where the corpse was stored	8 (100.00)	19 (46.34)	0.00(-)^3^	1.00		5.49	0.019^1^
Talked face to face	2 (25.00)	20 (48.78)	2.85(0.51–15.85)	0.23		0.82	0.36^1^
The index patient’s contaminated items	8 (100.00)	8(19.51)	0.00(-)^3^	1.00		15.77	0.00^1^
The index patient’s vomitus	1 (12.50)	0 (0.00)	0.00 (-)^3^	1.00		-	0.17^2^
The index patient’s excreta	1 (12.50)	1 (2.44)	0.18 (0.010–3.14)	0.24		-	0.31^2^
The index patient’s blood or body fluids	2 (25.00)	7 (17.07)	0.62 (0.10–3.72)	0.60		0.00	1.00^1^
Rats in the households	0 (0.00)	4 (9.76)	0.00 (-)^3^	1.00		-	1.00^2^
Domestic animals	2 (25.00)	5 (12.20)	2.40 (0.38–15.32)	0.36		0.13	0.71^1^
Weeds and shrubs in household	2 (25.00)	2 (4.88)	6.5(0.77–55.25)	0.086		-	0.12^2^
Wild animals in household or working place	1 (12.50)	5 (12.20)	1.03 (0.10–10.20)	0.98		0.00	1.00^1^
Worked in the field	6 (75.00)	23 (56.09)	2.35 (0.42–13.05)	0.33		0.28	0.60^1^
Weeds and shrubs in workplace	5 (62.50)	19 (46.34)	1.93 (0.41–1.96)	0.41		0.15	0.70^1^

^1^Continuity correction χ^2^ tests

^2^Fisher’s exact test

^3^Infifinite or not able to be calculated

## Discussion

SFTS is a common infectious disease that is transmitted primarily by tick bite [1]. SFTSV can also be transferred person-to-person through contact with blood, secretions or mucous of a patient with SFTS [2, 18, 23]. In this study, the Index patient had a history of exposure to ticks during farm work. When we examined the Index patient’s close contacts, 16.3% (8/49) were positive for virus-specific IgM, which is higher than those in other studies [24, 25]. Li et al. have reported the seroprevalence of SFTSV in healthy Chinese people varies from 0.23 to 9.17%, depending on the population, geography, as well as the test reagent and methods. Only a small proportion of exposed individuals develop clinical symptoms [26]. In this study, we also conducted screening on a healthy population in the village where the Index case occurred, the nucleic acid and antibody test results were negative for all the volunteers.

Studies have reported that new bunia virus IgM antibody of SFTS cases can convert from 5 to 15 days after the onset of disease. Researchers concluded that IgM antibodies to the new bunia virus was a possible indicator of infection and might be useful as an alternate method for the early diagnosis of SFTS [27, 28]. We carried out a case-control study to explore the association of exposure factors and SFTSV infection. The results found that the poor ventilation where the corpse (χ^2^ = 5.49, *P* = 0.019) and contacting the Index patient’s contaminated items (χ^2^ = 15.77, *P* < 0.001) were significantly related to SFTSV infection. Among the eight close contacts whose IgM antibodies were tested positive, one person was continuously exposed to the Index case from her SFTS onset to her funeral. Seven individuals had only one exposure during the period of the Index case’s funeral. Yoo et al. reported asymptomatic infections in family cluster of SFTS in Korea due to bloodless exposure [21]. Gu et al. reported an epidemiological investigation on an outbreak of severe fever with thrombocytopenia syndrome, they carried out a retrospective cohort study and found that direct contact with blood was an important risk factor, however, aerosol transmission could not be ruled out [29].

In Yantai city, some rural areas retain traditional funeral customs. When infected patients in the hospital are near death, the family would request discharge. And when patients die, they handle the funeral proceedings in the village. Both activities increase the risk of human-to-human infection and outbreak. In our study, the three secondary cases resided in different communities, and none of them reported a history of tick bite or animal contact. In addition, all three cases were diagnosed within the longest incubation period after contacting the Index case. Even if the contact history investigation was incomplete, they were less likely to be bitten by ticks at the same time. Case 1 and Case 2 exposed the blood of the Index patient with personal protection, and later remained in a closed space storing the patient’s body and her blood contaminated clothing for a long time. They may be infected with SFTSV through blood or aerosols. Case 3 had not directly contact with the blood of the Index patient but stayed in the room where the body and her blood contaminated clothing was stored for a long time. Thus, we speculated that he may be infected through aerosols. What’ more, the five IgM-positive cases did not directly contact the Index case and only attended the funeral without personal protective measures. They were detected positive during the longest incubation period after attending the funeral, so they may also be infected by SFTSV through aerosols in closed environment.

Our case-control study has some limitations. First, the sample size was relatively small considering the heterogeneity of the study participants. Second, we could not identify a dose-response relationship for frequency of exposure to the Index case and the risk of infection. Third, due to laboratory constraints, we were unable to test viral load in these cases.

## Conclusions

We infer that this cluster outbreak was caused by person-to-person transmission. Direct contact with the patient’s blood may cause SFTSV transmission. In addition, it can also be caused by aerosol in a poorly ventilated environment. The lesson from this cluster of possible person-to-person transmission of SFTSV is to have strict procedures in medical institutions for handling contaminated bodies of the deceased patients by special personnel. Bodies should be sealed in yellow bags and sent directly to a crematorium in order to reduce the risk of exposure to the virus.

## Data Availability

The datasets generated during and/or analysed during the current study are available from the corresponding author on reasonable request.

## Abbreviations

SFTS: Severe fever with thrombocytopenia syndrome
SFTSV: SFTS virus
ICTV: International Committee on Taxonomy of Viruses
L segment: large segment
M segment: medium segment
S segment: small segment
RdRp: RNA-dependent RNA polymerase
NP: nucleocapsid protein
NSs: nonstructural protein
IFN: interferon
RT-PCR: reverse transcription polymerase chain reaction
ORs: odds ratios
95% CI: 95% confidence intervals
WBC: white blood cell
CK: creatine kinase
LDH: lactase dehydrogenase
CT: computed tomography
